# Underwater Localization System Combining iUSBL with Dynamic SBL in ¡VAMOS! Trials

**DOI:** 10.3390/s20174710

**Published:** 2020-08-20

**Authors:** José Almeida, Bruno Matias, António Ferreira, Carlos Almeida, Alfredo Martins, Eduardo Silva

**Affiliations:** 1 INESC Technology and Science, Centre for Robotics and Autonomous Systems, 4200-465 Porto, Portugal; antonio.b.ferreira@inesctec.pt (A.F.); carlos.almeida@inesctec.pt (C.A.); alfredo.martins@inesctec.pt (A.M.); eduardo.silva@inesctec.pt (E.S.); 2 ISEP-School of Engineering, Electrical Engineering Department, 4200-072 Porto, Portugal

**Keywords:** AUV navigation, underwater localization, estimation, SBL, iUSBL, data fusion, EKF, underwater mining

## Abstract

Emerging opportunities in the exploration of inland water bodies, such as underwater mining of flooded open pit mines, require accurate real-time positioning of multiple underwater assets. In the mining operation scenarios, operational requirements deny the application of standard acoustic positioning techniques, posing additional challenges to the localization problem. This paper presents a novel underwater localization solution, implemented for the ¡VAMOS! project, based on the combination of raw measurements from a short baseline (SBL) array and an inverted ultrashort baseline (iUSBL). An extended Kalman filter (EKF), fusing IMU raw measurements, pressure observations, SBL ranges, and USBL directional angles, estimates the localization of an underwater mining vehicle in 6DOF. Sensor bias and the speed of sound in the water are estimated indirectly by the filter. Moreover, in order to discard acoustic outliers, due to multipath reflections in such a confined and cluttered space, a data association layer and a dynamic SBL master selection heuristic were implemented. To demonstrate the advantage of this new technique, results obtained in the field, during the ¡VAMOS! underwater mining field trials, are presented and discussed.

## 1. Introduction

Currently, underwater technologies constitute a major engineering topic, as exploration and exploitation tasks rely on unmanned underwater vehicles (UUVs) with some level of autonomy, capable of withstanding harsh environment conditions. The constant evolution in underwater robotics materializes in systems with greater levels of autonomy, able to cover larger areas, with higher accuracy and for longer deployment periods. In this context, the localization system assumes a major role, since knowledge about the vehicle’s position and orientation is crucial to support other tasks such as exploration, environment intervention, and mission planning.

**Figure 1:**
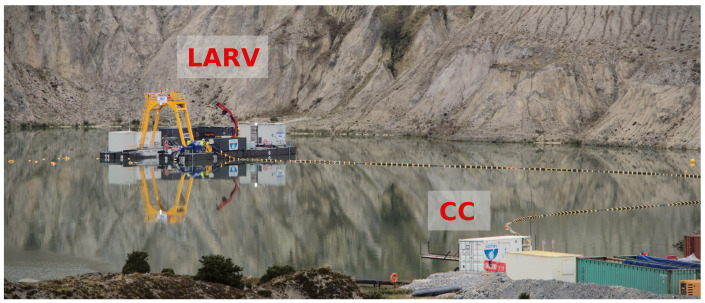
Picture taken during the ¡VAMOS! field trials, showing the launch and recovery vessel (LARV) and the control cabin (CC). At this point in time, the MV was deployed underwater.

**Figure 2:**
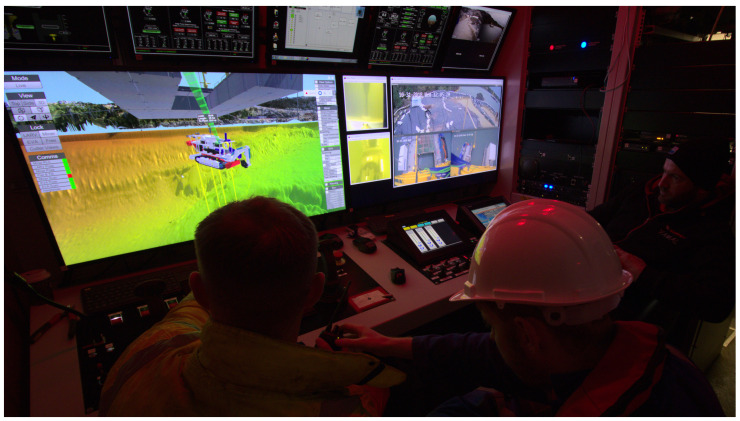
Team of pilots, inside the control cabin, performing the MV deploying maneuver, assisted by the virtual reality display.

This article covers the localization system developed for a mining vehicle, working on an underwater mining exploration inside open pit flooded mines (see Figure 1). The work was developed in the context of the ¡VAMOS! project, where an innovative approach for underwater exploitation of mineral resources was demonstrated successfully [1]. The ¡VAMOS! robotic team is composed of a remotely controlled mining vehicle (MV), a surface vessel to launch and recover the mining vehicle, and an exploration AUV called EVA. Together, all vehicles cooperate in the self-localization task, while collaboratively collecting geometric data of the underwater scenario. The mapping information feeds a 3D virtual environment, based on which a team of pilots remotely supervises and controls the mining operations ashore (see Figure 2). In this context, the quality of the localization estimate is essential not only to produce a faithful virtual reconstruction of the underwater environment, which is crucial to develop the entire mining operation, but also to ensure safe navigation inside the heavy cluttered space. Besides accuracy, system reliability was also of major concern when designing the localization strategy, as any failure could lead to a complete standstill of the mining operation and force a full restart of the entire system.

Keeping an accurate localization estimate, during long periods of submerged operation, poses a difficult challenge, as a result of severe degradation affecting artificial perception underwater. In this medium, localization references are characterized by poor resolution, a low sampling rate, and substantial noise perturbation. Therefore, long-term localization in six degrees of freedom can only be achieved by combining different sources of information [2]. Dead reckoning (DR) is the most widely used technique for underwater localization. It consists of a recursive method, in which relative displacement measurements are used to propagate the previous localization estimate. Those relative displacement observations are usually obtained from two sensors [3,4], namely a Doppler velocity log (DVL) and an inertial measurement unit (IMU). While the IMU provides high data rate linear acceleration references and angular velocity measurements, from which position and attitude estimates are obtained from integration, the DVL feeds direct velocity observations, with bounded uncertainty. These two information sources are combined in a probabilistic way, resorting to well established estimation methods, such as the Kalman filter (KF) or the particle filter (PF). The use of probabilistic estimation frameworks to perform data fusion provides the possibility to jointly estimate and compensate measurement bias, in order to minimize the impact of measurement error in the overall localization accuracy [3]. Despite this possibility and considering the integrative nature of dead reckoning techniques, the estimation uncertainty grows without bounds as a function of time. Hence, additional references must be considered in order to keep localization uncertainty within acceptable limits.

Direct position observations, retrieved from acoustic positioning systems [5], provide a valuable contribution to this objective [6]. The acoustic positioning technique involves the deployment of an array of acoustic beacons, with the objective of determining the relative position to a given target, equipped with an acoustic transponder. The position is computed based on the multilateration principle, which involves the exchange of acoustic pings between the beacon array and the transponder. In order to establish an acoustic positioning network, at least three beacons are necessary. Normally, acoustic positioning systems are characterized in terms of baseline length, i.e., the relative distance between elements of the beacon array. Three categories are usually considered, from long baseline (LBL) networks, with baselines in the kilometer range, passing to short baseline (SBL) modalities, using array elements placed several meters apart, to ultrashort baseline (USBL) configurations, with a few centimeters of displacement between array elements. Generally, positioning accuracy relates proportionally with baseline distance; however, the implementation of LBL and SBL networks poses additional challenges, either in terms of installation, calibration, and recovery effort [7] or in terms of system acquisition costs. For those reasons, USBL configurations are commonly associated with UUV operations.

The flooded open pit mine scenario presents new challenges, not common in the traditional open sea environment. From the acoustic positioning perspective, issues such as acoustic reflections and multipath in the confined and cluttered environment, occlusions resulting from the presence of risers and umbilical cables, and acoustic noise caused by the mining equipment and sound velocity variations, as a result of plume and turbidity changes, need to be addressed. From the operational point of view, all efforts related to the deployment, setup, and maintenance of the acoustic support system were also taken into account in the system design phase. Therefore, the use of bottom beacons or floating beacons was considered to be disadvantageous. Bottom beacons were avoided, since the mining operation itself produces a morphological change in the bottom, and beacons would eventually need to be relocated, with a huge logistic impact. The use of floating beacons installed on buoys was also discarded, considering their incompatibility with other equipment, including the floating part of the riser system. Since the mineral vein can extend for hundreds of meters, the working area covers the entire mine area up to a depth of 500 m.

The major contributions of this article are associated with the acoustic positioning system. The acoustic network is composed of an array of three transponders, mounted on board the launch and recovery vessel (LARV), to establish an SBL array with fixed baselines. Each underwater vehicle, namely the MV and EVA, are equipped with USBL transponders, in a configuration known as inverted USBL (iUSBL), as the USBL transponder is assembled on board the target. Considering that the positioning measurement is computed at the array side, the iUSBL configuration makes the positioning references readily available on board the UUV, facilitating its integration into the data fusion stream, with minimal time delay [8,9]. This overall configuration allows positioning references to be computed on both ends, i.e., the SBL array can track all underwater targets, while each underwater vehicle is able to localize itself with respect to the surface vessel using the iUSBL transponder.

This article investigates further the possibilities opened by the iUSBL configuration, in particular the ability to work with raw iUSBL measurements in the polar coordinate system, to directly observe the UUV’s heading angle. The ability to retrieve a heading observation from the acoustic positioning system not only enables the correction of the heading angle drift, which translates into longer deployment periods by aiding the north keeping capabilities of the gyro system, but also allows the localization system to be fully initialized underwater. The possibility of retrieving heading references and simultaneously compute position measurements from the iUSBL transponder constitutes a clear advantage of our iUSBL implementation. Moreover, by modeling each incoming ping using the polar coordinate system, in situations where the trilateration method fails to compute a positioning measurement, due to the loss of the signal from one or two SBL beacons, it is still possible to retrieve some information even when only a single SBL ping is received at the iUSBL side.

A strategy to dynamically assign the role of the master beacon in the SBL array is also purposed. The goal is to optimize the acoustic network geometry in order to maximize the observability of the heading angle at the iUSBL transponder side. On the other hand, by analyzing the measurement innovation within the Kalman filter framework, it is possible to identify SBL beacons subjected to multipath and occlusion. This information is also taken into account when selecting the master beacon from the SBL array.

The paper is organized as follows: In the next section, an overview of the ¡VAMOS! project is given, and the acoustic localization system is presented. In Section 3, the localization method is detailed. The novel underwater localization system integrating iUSBL and SBL is thoroughly presented along with the dynamic acoustic master selection method. Field trial results in the operational scenario are reported and discussed in Section 4. Finally, a set of conclusions and the next steps are enunciated in Section 5.

## 2. ¡VAMOS! System Architecture

One major goal of the ¡VAMOS! project was to demonstrate the possibility of mining open pit flooded mines, using a team of semi-autonomous machines. The complete setup was designed to support the deployment and operation of a 30 ton crawler excavator, the mining vehicle (MV), which by means of a front cutter head and a back digging bucket, collects ore to be pumped to the surface, through a riser system. A surface barge, referred to as the launch and recovery vessel (LARV), serves as an interface between the MV and the control cabin (CC) on shore (see Figure 1). Besides the ability to mechanically deploy and recover the MV from the bottom of the mine, the surface barge also supplies the MV with electrical power, establishes an SBL acoustic positioning array, and self-localizes using a triple antenna GNSS system [1,10]. The CC constitutes the operations center, where pilots drive the MV and the LARV inside a virtual reality environment. This virtual representation of the underwater scenario is constantly being updated, as the mining work progresses, based on information collected by the sensors on board the MV itself and further enriched with data provided by EVA, a surveying AUV.

### 2.1. Communication and Synchronization Networks

**Figure 3:**
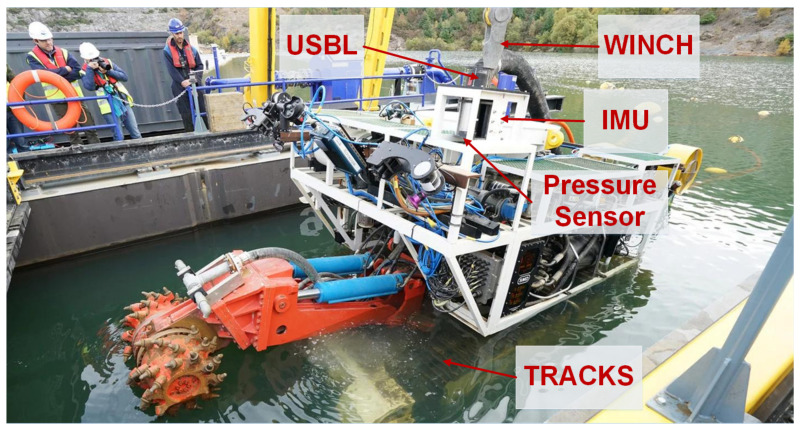
Localization sensors installed on board the mining vehicle.

The main computational effort is devoted to building the virtual environment representation and accurately localizing all vehicles inside that model. Apart from some residual local processing on board the LARV, related to the triple antenna GNSS system, all remaining data processing is performed inside the CC. Data coming from sensors on board the MV (See Figure 3) reach the CC directly through a fiber optic umbilical cable, while sensor data collected on the LARV are transmitted over another fiber optical channel. In this way, the localization computer, inside the CC, collects all sensor information from the LARV and the MV, in order to estimate the localization of both platforms.

**Figure 4:**
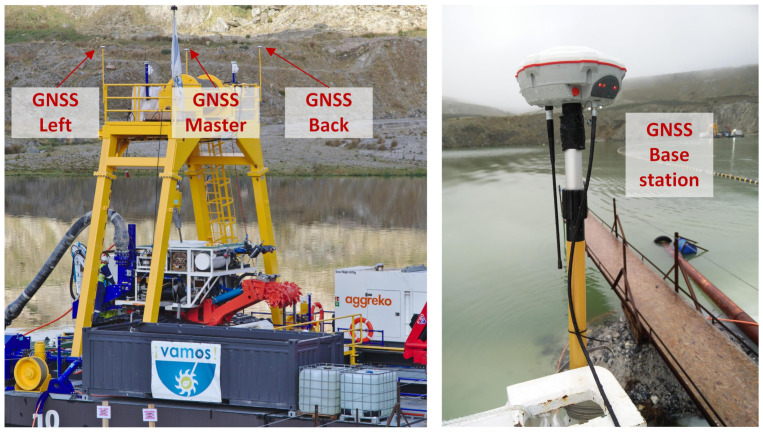
GNSS positioning network, composed of a triple antenna receiver on board the LARV and a fixed base station on shore. Synchronization references for the LARV’s systems are supplied by the GNSS receivers on board, while time references for synchronizing CC and MV equipment are provided by the base station.

Since acoustic positioning involves time of flight measurements, keeping a common time reference is essential to ensure that the position can be computed at every node of the acoustic network. Synchronization references, obtained from GNSS receivers, are broadcast to all devices. In this way, a consistent time reference is maintained between all computers and sensors. The base station ashore (see Figure 4) provides synchronization references, in the form of a pulse per second (PPS) plus NMEA sentences, for every equipment inside the CC and on board the MV. For the MV, time references are fed through the umbilical cable. Similarly, on board the LARV, synchronization references obtained from the triple antenna GNSS system are supplied to the SBL network. Each SBL transponder runs a custom firmware, offering the capability of adjusting its internal clock based on the synchronization references received every second.

### 2.2. Acoustic Localization Network in the ¡VAMOS! Project

The characteristics of the ¡VAMOS! scenarios raise some unusual concerns, when designing an acoustic positioning network. The installation of a beacon array on the bottom, according to an LBL configuration, is not a feasible option, considering the impact of the mining process on the underwater morphology. Beacons would eventually need to be relocated, as the mining vehicle approaches the installation. Assembling the array on the surface, using buoys, is not an alternative, since the water surface must be as clear as possible for the LARV to move. Additionally, buoys would become incompatible with other floating hardware, such as pumping tubes, umbilical and communication cables, and anchor wires, which stand loose at the surface.

The decision was made to assemble an SBL network, composed of three beacons, on board the LARV. Besides reducing the amount of hardware inside the water basin, such a configuration ensures an appropriate coverage of the mining area, as the MV operates mostly below the LARV. Additionally, the SBL array is accurately positioned in the global frame, based on the LARV’s localization system, composed of a tipple antenna GNSS system [1,10] (see Figure 4 and refer to Table 1 for a description of the GNSS receivers). The acoustic measurements coming from the SBL network and the MV’s iUSBL are processed by the main localization computer inside the CC. Those measurements are fused with data from other sensors, using an extended Kalman filter, as described in the next section.

The acoustic network is formed by Evologics transceivers, which offer a wide beam pattern of 100 degrees (for detailed specifications, see Table 1). This product range has a built-in acoustic modem function, allowing the user to encode short messages in the acoustic pings. Hence, information can be exchanged between acoustic nodes while simultaneously performing positioning.

The SBL array is installed on board the LARV in an L-shaped configuration. In a preliminary step, the entire array is calibrated using a GNSS based technique, in which antennas are placed in the surface extension of each transceiver mounting pole. From this procedure, the relative positions of each transceiver are determined with respect to the LARV’s body frame with high accuracy.

From this point forward, a distinction is made between the master beacon in the SBL array and the remaining transponders. The master beacon is the one responsible for performing the interrogation of the acoustic network. Hence, every second, the master beacon sends an acoustic ping, to which target vehicles reply immediately using their iUSBL transponders. At this point, localization observations are computed at the iUSBL side. The iUSBL responses are listened to by the elements of the SBL array, in order to track the position of the underwater targets.

## 3. Localization System

**Figure 5:**
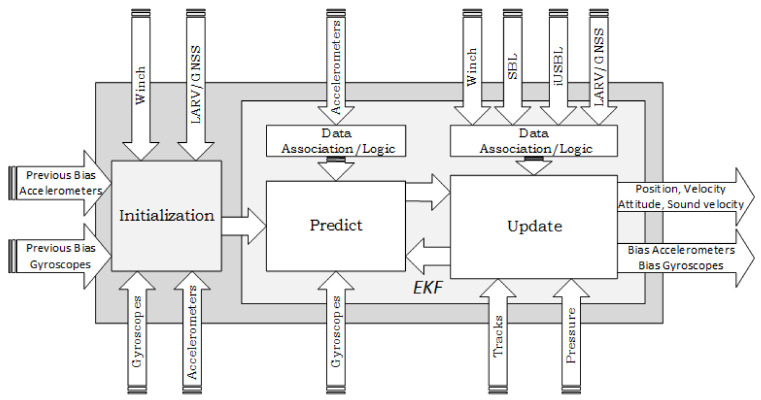
Architecture of the localization method developed for the MV.

Conceptually, the localization system developed for the MV follows the architecture depicted in Figure 5. After an initialization routine, where the initial values for the localization states are determined, the recursive cycle of the EKF is performed, by executing the predict/update loop. The estimation method computes the MV localization solution, composed of the position, velocity, and attitude. Additionally, the IMU bias states are also estimated and compensated, to improve the dead reckoning performance. The sound velocity parameter, which directly affects the acoustic range measurements, is also estimated in real time.

Following Figure 5, the localization procedure starts by running an initialization routine, to determine the initial values for the localization states. This step was conceived of to be executed both outside or inside water. Initializing above water becomes difficult, as the MV sensor set was selected to mainly perform underwater. As a result, the capability to localize above water is very limited; hence, only a coarse initialization is possible. Nonetheless, performing this coarse initialization is important to provide awareness information to the operators inside the CC, to assist in the diving maneuver.

If the accelerometer and gyroscope bias have been estimated, during a previous dive, the last estimates are loaded, otherwise those states are initialized to zero. For the velocity states, as the LARV keeps a fixed position during the deployment maneuver, velocity is initialized to zero. If a previous estimate for sound speed is available, it is also loaded, or else, a measurement from an external sound velocity probe (SVP) is supplied. To perform attitude initialization, the MV should remain stationary, either at the LARV’s deck or at the bottom of the mine. The initial roll and pitch angles are computed indirectly, by measuring the components of the gravity vector using the accelerometers [11]. The heading angle is determined through a north seeking procedure, which is not under the scope of this article. Finally, position initialization follows different methods, depending on if the MV is above or under water. While above water, there is no sensor to directly measure the MV’s position. In this case, the initialization of the position states is accomplished by considering that, when suspended by the crane, the MV behaves as a pendulum, remaining aligned with the winch cable. Following this assumption, the LARV’s position is transferred to the MV considering the cable length released by the crane system. When the MV is initialized underwater, the acoustic positioning system is used to determine the initial position.

After initialization, a dead reckoning strategy is used to predict the position, attitude, and velocity, based on raw measurements from the IMU. The dead reckoning solution is corrected in the update stage of the EKF, by considering measurements from several sources (See Figure 3). Through the iUSBL, position and orientation observations are obtained with respect to the SBL array. Nonetheless, by considering the LARV’s localization, relative measurements obtained by means of the iUSBL system are transformed into global position and orientation corrections. Considering the permanent connection between the LARV and the CC, through a fiber optical cable, the SBL measurements are always available for integration in the MV’s localization solution. Furthermore, a pressure sensor provides direct depth observations. Odometry information, coming from the track locomotion system, is used to correct velocity states. While the MV is suspended by the cable, information about the winch system, combined with the LARV localization, is also considered. The technical specifications of the navigation system components are presented in Table 1.

### 3.1. Motion Model

In order to simplify notation, we assume the vehicle body frame (*b*-frame) to be coincident with the IMU reference frame (forth-right-down (FRD)), denoted by *b*. The navigation frame *n* corresponds to the local level navigation frame (*n*-frame) (north-east-down (NED)). The Earth frame (*e*-frame) is denoted by *e*, and *i* defines the inertial frame (*i*-frame).

The prediction model uses standard inertial mechanization equations (Equations (1) to (3)) to propagate the attitude, velocity, and position by performing inertial navigation in direct mode [11,12].

(1) $${\dot{q}}_{b}^{n}=\frac{1}{2}q_{b}^{n}⊗\omega_{nb}^{b}$$

(2) $${\dot{v}}^{n}=C_{b}^{n}f^{b}-\left(2\omega_{ie}^{n}+\omega_{en}^{n}\right)\times v^{n}+g^{n}$$

(3) $$\dot{p}=R_{p}v^{n}$$

The quaternion parameterization of the MV’s attitude is defined as $q_{b}^{n}=\left[q_{0}\;\;\rho \right]$ with a scalar component $q_{0}$ and a three element vector part $\rho =[q_{1}\;q_{2}\;q_{3}]$. The operation represented by ⊗ corresponds to a quaternion multiplication. $\omega_{nb}^{b}$ indicates the body angular rate with respect to the navigation frame. A rotation matrix $C_{b}^{n}$ from the *b*-frame to the *n*-frame is obtained from the quaternion as follows:

(4) $$C_{b}^{n}=\left[\begin{array}{ccc} q_{1}^{2}+q_{2}^{2}-q_{3}^{2}-q_{4}^{2} & 2(q_{2}q_{3}-q_{1}q_{4}) & 2(q_{1}q_{3}+q_{2}q_{4})\\ 2(q_{2}q_{3}+q_{1}q_{4}) & q_{1}^{2}-q_{2}^{2}+q_{3}^{2}-q_{4}^{2} & 2(q_{3}q_{4}-q_{1}q_{2})\\ 2(q_{2}q_{4}-q_{1}q_{3}) & 2(q_{1}q_{2}+q_{3}q_{4}) & q_{1}^{2}-q_{2}^{2}-q_{3}^{2}+q_{4}^{2} \end{array}\right]$$

The vector $v^{n}=\left[\begin{array}{ccc} v_{N}^{n} & v_{E}^{n} & v_{D}^{n} \end{array}\right]$ expresses the vehicle velocity in the local navigation frame. $f^{b}$ defines the specific force vector measured by the accelerometers. The gravity vector is represented by $g^{n}$. × denotes the cross product between the two vectors. $p=\left[\begin{array}{ccc} \phi & \lambda & h \end{array}\right]$ represents the geodetic position in terms of latitude ϕ, longitude λ, and height *h*. Matrix $R_{p}$ defines the local Earth curvature by:

(5) $$R_{p}=\left[\begin{array}{ccc} \frac{1}{R_{M}+\hat{h}} & 0 & 0\\ 0 & \frac{1}{\cos\left(\hat{\phi }\right)\left(R_{N}+\hat{h}\right)} & 0\\ 0 & 0 & -1 \end{array}\right]$$

where $R_{M}$ is the radius of the Earth and $R_{N}$ is the prime vertical radius of curvature.

The gyroscope measurements are modeled as the combination of the turn rate $\omega_{ib}^{b}$, plus a gyroscope bias term $\nabla^{b}$ and a zero-mean Gaussian white-noise term $\eta_{gv}$:

(6) $$\begin{array}{c} {\tilde{\omega }}_{ib}^{b}=\omega_{ib}^{b}+\nabla^{b}+\eta_{gv}\\ \hat{\nabla^{b}}=\eta_{gu} \end{array}$$

The accelerometer measurement model follows a similar representation:

(7) $$\begin{array}{c} \tilde{f^{b}}=f^{b}+\xi^{b}+\eta_{av}\\ \hat{\xi^{b}}=\eta_{au} \end{array}$$

with an accelerometer bias term $\xi^{b}$, $\eta_{au}$ and $\eta_{av}$ being zero-mean Gaussian white-noise random variables.

Finally, the speed of sound in water is modeled as a zero-mean Gaussian white-noise variable:

(8) $$\hat{sv}=\eta_{sv}$$

Therefore, through Equations (1)–(3) and (6)–(8), the state vector of the filter system can be defined as:

(9) $$x_{t}={\left[\begin{array}{cccccc} p & q_{b}^{n} & v^{n} & \nabla^{b} & \xi^{b} & sv \end{array}\right]}^{T}$$

An extended Kalman filter was implemented in order to accommodate the non-linear equations:

(10) $$\begin{array}{c} x_{t}=f\left({\hat{x}}_{t},u_{t}\right)+w_{t}\\ F_{t}=\frac{\partial f}{\partial x}|{\hat{x}}_{t},u_{t} \end{array}$$

where f(.) denotes the filtering prediction model and $w_{t}∼N\left(0,Q_{t}\right)$ the process white Gaussian noise in discrete time with zero mean $Q_{t}$. $F_{t}$ is the Jacobian matrix of the non-linear model function f(.) with respect to the states $x_{t}$.

### 3.2. Acoustic Localization System

The dead reckoning strategy, developed during the prediction step, is characterized by unbounded uncertainty growth, as a result of the consecutive integration of measurement errors [13]. In this context, periodic global measurements from the acoustic positioning network are essential to ensure that the localization estimate remains accurate, during long operation periods.

**Figure 6:**
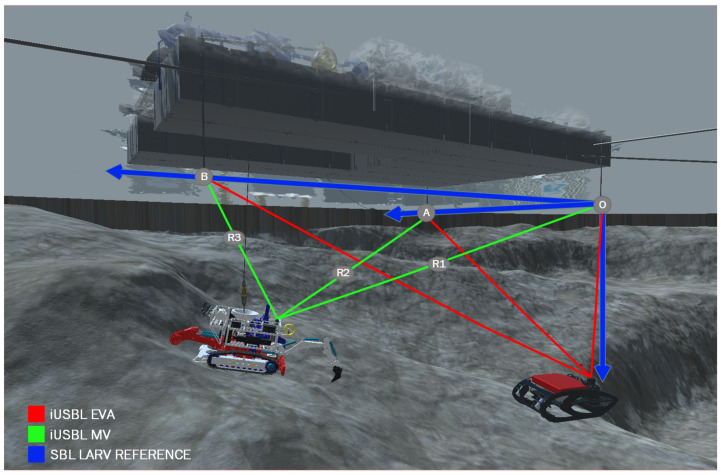
Interaction between the SBL network on the LARV and the iUSBL on the MV and EVA.

The SBL array is installed on board the LARV in an L-shaped configuration, as shown in Figure 6, forming two orthogonal baselines. The origin of the SBL array is located at the transceiver O, and the SBL reference frame is oriented with the X-axis pointing in the direction of the transponder B, while the Y-axis points in the direction of the transponder A.

#### 3.2.1. Range Based Observation Model

Traditional SBL systems use slant range measurements to perform positioning using the trilateration method [14]. Usually, a beacon is selected to be the master, whose role is to periodically, typically every second, interrogate targets equipped with transponders, which should reply back to the SBL array. Hopefully, the response from the UUV can be listened to by at least three SBL beacons. Assuming the position of the three beacons is precisely known, through a prior calibration procedure, the target position can be calculated by the slant ranges $r_{i}$, which are given by:

(11) $$r_{i}=\frac{sv\;\;\tau_{i}}{2}$$

where sv denotes the speed of sound on the water column and $\tau_{i}$ is the propagation time, i.e., the time the acoustic signal takes to travel from the master to the target transceiver plus the time for the reply to reach back to the *i*th SBL node. Considering a three beacon SBL array, from the three slant ranges $r_{1:3}$, forming the baselines *a* and *b*, the position of a given target, in Cartesian coordinates x,y,z, can be obtained using:

(12) $$x=\frac{r_{1}^{2}-r_{2}^{2}+a^{2}}{4a}$$

(13) $$y=\frac{r_{1}^{2}-r_{3}^{2}+b^{2}}{4b}$$

(14) $$z=\frac{\sqrt{r_{1}^{2}-x^{2}+y^{2}}+\sqrt{r_{2}^{2}-{\left(x-a\right)}^{2}+y^{2}}+\sqrt{r_{3}^{2}-x^{2}+{\left(y-b\right)}^{2}}}{3}$$

As Equations (12)–(14) demonstrate, the echo needs to reach three SBL transceivers for a position to be computed. Moreover, the speed of sound must be known in advance in order to calculate slant ranges (Equation (11)). For a unique position to be computed, all three slant ranges should perfectly agree, in order to intersect at a single point. However, error sources caused by the environment conditions, internal sensor noise, inaccurate calibration, and speed of sound variations introduce perturbations to the measured ranges, leading to an uncertainty about the true target position. Hence, optimization techniques are applied to this problem [15].

Instead, our solution proposes the direct integration of slant range measurements into the data fusion filter. By doing so, information from individual ranges is still useful when less than three echos are received at the SBL side, resulting in a more robust, flexible, and scalable method. This technique is particularly useful in the ¡VAMOS! context, where occlusions and acoustic noise may affect the reception of echos by the SBL array. Furthermore, as the calculation of slant ranges depends on the speed of sound, we developed our observation model to be able to estimate this parameter, in an indirect manner, which is estimated by the EKF.

The observation is formulated in terms of slant range, computed according to Equation (11), multiplying the time of flight (TOF) measurement by the speed of sound sv, estimated by the EKF. The predicted observation, at time *t* with respect to the $i^{th}$ SBL transceiver, is obtained through the observation function $h_{i}^{SBL}$.

(15) $$\begin{array}{c} h_{i}^{SBL}\left(x_{t},\nu_{i}\right)=\frac{\sqrt{{\left(b_{x_{i}}-p_{x}\right)}^{2}+{\left(b_{y_{i}}-p_{y}\right)}^{2}+{\left(b_{z_{i}}-p_{z}\right)}^{2}}}{sv}+\nu_{i} \end{array}$$

where $p_{t}={\left[p_{x},p_{y},p_{z}\right]}^{T}$ indicates the position of the target iUSBL in the n-frame at time *t*. $b_{i}={\left[b_{x_{i}},b_{y_{i}},b_{z_{i}}\right]}^{T}$ defines the position of the $i^{th}$ SBL beacon in the navigation reference frame, and $\nu_{i}∼N\left(0,R_{i}\right)$ indicates the noise affecting the observation, with a zero mean white Gaussian noise $R_{i}$.

$H_{i}^{SBL}$ represents the Jacobian matrix, which linearizes the nonlinear measurement function $h_{i}$ around the predicted state estimate ${\hat{x}}_{\left(t|t-1\right)}$:

(16) $$\begin{array}{c} H_{i}^{SBL}=\frac{\partial h_{i}}{\partial x}|{\hat{x}}_{\left(t|t-1\right)}=\\ =\left[\begin{array}{ccccc} \frac{-2bi_{x}+2p_{x}}{2c} & \frac{-2bi_{y}+2p_{y}}{2c} & \frac{-2bi_{z}+2p_{z}}{2c} & O_{1\times 13} & -\frac{c}{sv^{2}} \end{array}\right] \end{array}$$

where $c=\sqrt{b_{x_{i}}^{2}-2b_{x_{i}}p_{x}+p_{x}^{2}+b_{y_{i}}^{2}-2b_{y_{i}}p_{y}+p_{y}^{2}+b_{z_{i}}^{2}-2b_{z_{i}}p_{z}+p_{z}^{2}}$.

The size of the observation vector *h*, Jacobian matrix *H*, and covariance matrix *R* changes according to the number of echos received by the SBL array at each time step *t*.

Therefore, two main advantages can be identified in relation to traditional multilateration methods. Our solution is able to aid localization even when a single SBL transceiver listens to an echo. Secondly, it allows indirect estimation of the average speed of sound along the water column, resulting in real-time corrections of this parameter in order to increase slant range observation accuracy.

#### 3.2.2. iUSBL Observation Model

In the ¡VAMOS! scenario, local disturbances affecting the magnetic field make magnetometer measurements inaccurate. Therefore, the determination of the heading angle is achieved through the integration of gyroscope measurements. Even with high performance fiber optic gyroscopes (FOG), alternative direct observations are necessary to mitigate drift affecting the heading angle. One novel solution of our work consists of retrieving relative orientation observations from the iUSBL system, by analyzing the direction of incoming pings sent from the master beacon. This not only ensures a correct heading estimate during long operation periods, but also enables full attitude initialization underwater, without resorting to north seeking approaches.

**Figure 7:**
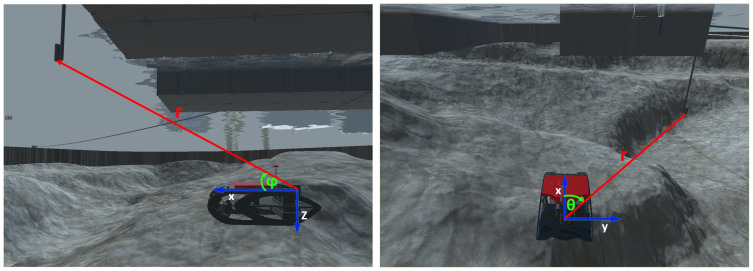
Illustration of the polar observation obtained through the iUSBL: (**a**) elevation angle and range; (**b**) bearing angle and range.

By adopting a polar representation, directional angles between the iUSBL antenna and the SBL master beacon *i* are readily available. Through time or phase differences of arrival, measured at the iUSBL array [16], polar measurements, parameterized in terms of an horizontal angle, denoted as bearing $\theta_{i}$, and a vertical elevation angle $\varphi_{i}$ are retrieved with respect to the master SBL beacon, as illustrated in Figure 7. Further, the adoption of a synchronized interrogation technique allows the determination of the relative distance, range ($r_{i}$), based on the time of flight of each ping measured by the iUSBL. Usually, a conversion to the Cartesian coordinate system is performed to calculate the displacement of the master beacon *i* with respect to the iUSBL, as follows:

(17) $$\begin{array}{c} x=r_{i}\cos\left(\varphi_{i}\right)\cos\left(\theta_{i}\right)\\ y=r_{i}\cos\left(\varphi_{i}\right)\sin\left(\theta_{i}\right)\\ z=r_{i}\sin\left(\varphi_{i}\right) \end{array}$$

The inverse conversion can also be defined, to translate Cartesian coordinates to the polar coordinate system, as:

(18) $$\begin{array}{c} \varphi_{i}=\arccos\left(z/r_{i}\right)\\ \theta_{i}=\arctan\left(y/x\right)\\ r_{i}=\sqrt{x^{2}+y^{2}+z^{2}} \end{array}$$

Since we chose to work with raw measurements in the polar coordinate form, the iUSBL observation with respect to beacon *i*, expressed with respect to the body frame, becomes:

(19) $$z_{i}={\left[\varphi_{i}\;\theta_{i}\;r_{I}\right]}^{T}$$

Considering $b_{i}^{n}$ the position of the $i^{th}$ beacon with respect to the navigation frame, the relative displacement in the MV’s body frame, expressed in Cartesian coordinates, is obtained by:

(20) $$p_{b}={\left(C_{b}^{n}\right)}^{T}\left(b_{i}-p\right)$$

Following Equation (18), a predicted observation can be obtained by converting Equation (20) to the polar coordinate system:

(21) $$h_{i}^{iUSBL}\left(x_{t},b_{i}\right)=\left[\begin{array}{c} {\hat{\varphi }}_{i}\\ {\hat{\theta }}_{i}\\ {\hat{r}}_{i} \end{array}\right]=\left[\begin{array}{c} \arccos(p_{b_{z}}/r_{i})\\ \arctan(p_{b_{y}}/p_{b_{x}})\\ \left|\right|p_{b}\left|\right| \end{array}\right]$$

The Jacobian matrix $H_{i}^{iUSBL}$, which linearizes the nonlinear measurement function, is given by:

(22) $$H_{i}^{iUSBL}=\frac{\partial h_{i}^{iUSBL}}{\partial x}|{\hat{x}}_{\left(t|t-1\right)}$$

As the observation model (Equation (21)) depends on position and attitude states, this set of localization states is subjected to correction during the update step to the EKF. Therefore, iUSBL measurements are not only used for positioning, but also to increase attitude estimation accuracy.

Nevertheless, considering that USBL systems concentrate all elements of the acoustic array in a small area, they become more susceptible to the multipath reflections and occlusions, increasing the risk of outliers. The dynamic master strategy, described next, helps to reduce periods of occlusion and multipath; however, even just a single outlier can potentially lead to filter divergence. To prevent this, a data association layer was developed to reject outliers. It relies on the Mahalanobis metric, given by:

(23) $$D_{ij}={{\tilde{y}}_{ij}}^{T}{S_{ij}}^{-1}{\tilde{y}}_{ij}<\chi_{d,\alpha }^{2}$$

where *d* is the dimension of the observations vector, α the desired confidence level of the chi-squared distribution $\chi^{2}$, and ${\tilde{y}}_{j}$ the innovation of the $j^{th}$ observation, and its associated covariance matrix $S_{j}$ are obtained as:

(24) $$\begin{array}{c} {\tilde{y}}_{j_{t}}=z_{j_{t}}-h_{j}\left({\hat{x}}_{t|t-1}\right)\\ S_{j_{t}}=H_{j_{t}}P_{t|t-1}{H_{j_{t}}}^{T}+R_{j} \end{array}$$

Finally, if the measurements are accepted, they will be used to update the state estimate by means of the EKF update equations:

(25) $$\begin{array}{c} K_{j_{t}}=P_{t|t-1}{H_{j_{t}}}^{T}{S_{j_{t}}}^{-1}\\ {\hat{x}}_{t|t}+K_{j_{t}}{\tilde{y}}_{j_{t}}\\ P_{t|t}=\left(I-K_{j_{t}}H_{j_{t}}\right)P_{t|t-1} \end{array}$$

#### 3.2.3. Dynamic Master Selection

In conventional SBL systems, once an acoustic transponder is defined as the master, the array operates according to that definition, and a change in master usually requires a complete system restart. However, in some scenarios, in particular the ones experienced in the ¡VAMOS! project, changing the master beacon during operation might become desirable, in an effort to improve state observability and also to mitigate occlusions and multipath reflections. Take for example the case of an occlusion, where the line of sight between the UUV and the master becomes disrupted by an object. In that situation, a different element in the SBL may offer a clear line of sight; hence, changing the master role becomes beneficial. Additionally, poor measurement consistency may also be associated with multipath, so changing the master beacon may also help. Improving state observability, in particular for the case of direction angle measurements of iUSBL systems, constitutes another motivation to perform the dynamic master selection, as pings coming from low elevation angles provide higher information about the heading angle.

**Figure 8:**
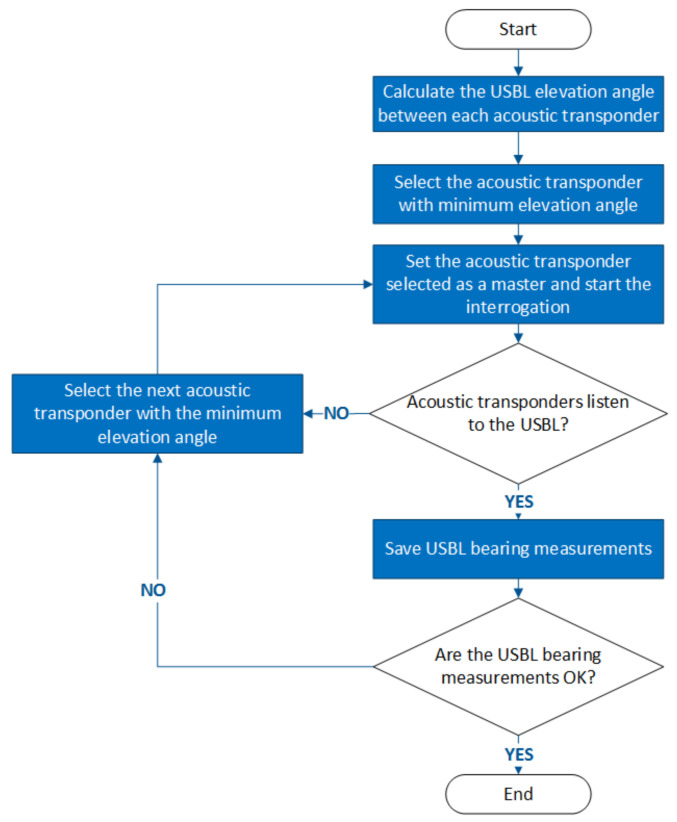
Flowchart of the dynamic master selection algorithm.

Therefore, a heuristic method depicted in Figure 8 was implemented in order to manage the online selection of the master beacon. At first, a specific beacon is selected and starts the interrogation process. The decision to select a different master is supported by the following criteria: to improve observability, the SBL beacon offering the minimum elevation angle is selected; in the case of occlusion, a different beacon should be selected; in the case of measurement inconsistency, associated with observations showing high innovation within the EKF, the master should also be replaced.

## 4. Results

The results presented in this section are based on real data acquired during the ¡VAMOS! field trials, conducted at Silvermines in the Republic of Ireland, during September and October of 2018. The LARV localization system, based on RTK-GNSS, will be used as the ground truth to evaluate the accuracy of the mining vehicle localization estimate during the recovery phase. This maneuver is performed once the mining operation is concluded and the MV is hoisted back to the surface. For the procedure to be successful, the LARV must be vertically aligned with respect to the MV, so that the winch system can attach to the MV. The margin for error is below 1 m. This value must not be exceeded, otherwise the recovering maneuver fails, jeopardizing the entire project. In fact, results show that the performance of our localization solution is much better, even for long deployment missions. Analyzing the localization performance at recovery time makes sense, as it coincides with the end of the MV’s mission. Moreover, at the moment the MV becomes suspended by the winch, considering its 20 ton weight in water, it becomes perfectly aligned with the LARV’s crane. A precise indication of depth is also available, as the length of the unwound cable is measured by the winch system. During this operation, our analysis concentrates mainly on checking the consistency of the localization estimation process, assessed by analyzing the innovation sequence of EKF. The innovation is an important measure of the deviation between filter estimates and observations [17], through which the filter consistency can be analyzed.

**Figure 9:**
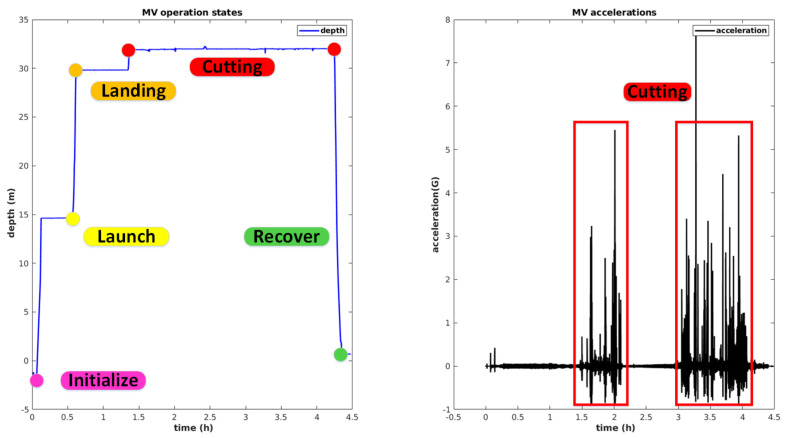
Summary of the mining vehicle mission: (**a**) representation of several mission phases; (**b**) acceleration values registered during the cutting phase.

The real dataset analyzed here consists of a full dive, with a duration of 4 h and 30 min, where the MV is lowered to the bottom, to a depth of 39 m. The complete mission, illustrated in Figure 9a, is composed of the following maneuver sequence:

- Initialization: At this stage, the MV stands stationary at the LARV’s deck, and the process of initializing the localization states begins, by performing the north seeking procedure and determining the roll and pitch angles through the observation of the gravity vector. Bias and sound velocity states are initialized at this point. After, the MV is suspended by the winch to initialize the position and velocity.
- Launching: After completing the initialization step, the MV is ready to be deployed in the water. The launching maneuver is performed by the action of the winch. Therefore, the position and velocity states receive updates from the winch observations until the acoustic system and pressure sensor start to provide measurements.
- Landing: In this state, all sensor measurements are available to be fused by the EKF, and so, the winch observations are discarded.
- Cutting: After landing, the winch is disconnected, and the MV is now free to move. At this point, odometry information from the tracks starts to be considered. The MV moves slightly, descends to engage a rocky bank, and starts cutting. When the MV is cutting, the gravity observation model is disabled, as high vibrations start to affect the accelerometer measurements, with accelerations up to 8G (see Figure 9b)), making the observation of the gravity vector impossible.
- Recovery: In this state, the MV moves away from the mining location, and after, the LARV aligns vertically. The winch attachment is lowered to establish the mechanical connection with the MV. The MV is finally hoisted back to the LARV’s deck.

To evaluate the performance of our contribution, both acoustic localization methods, namely the range based observation model and the iUSBL observation method, are evaluated in an isolated manner, in order to pinpoint the advantages of each contribution. Finally, the ¡VAMOS! localization solution is analyzed as a whole, taking advantage of the two observation methods. The estimation consistency is characterized in terms of the innovation sequence.

### 4.1. Range Based Observation Model Results

According to our method, the time of flight measurements registered at each SBL beacon are converted into slant ranges and integrated in the data fusion stream directly. In this way, individual measurements, collected at each node, can be used to aid positioning, even when the acoustic iUSBL reply is not listened to by the entire SBL array. Furthermore, since the observation depends on the speed of sound, they contribute to estimating this parameter in an indirect manner.

**Figure 10:**
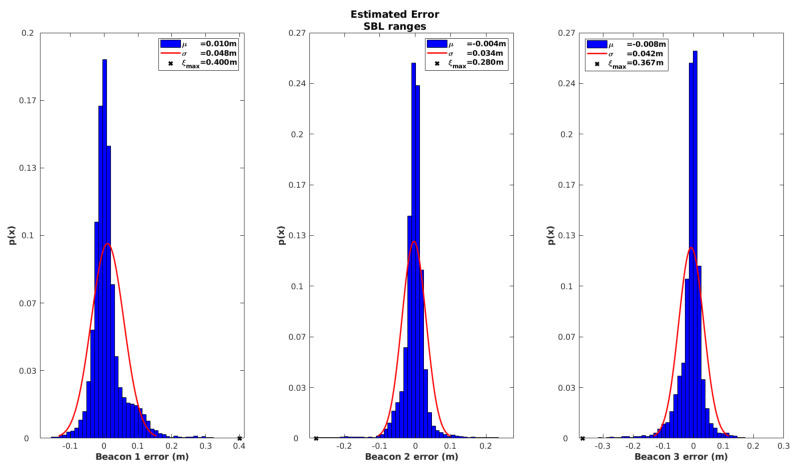
Innovation statistics registered for the observations of each SBL beacon, using the range based model.

Figure 10 characterizes the innovation associated with the observations of each SBL beacon, in the form of a histogram. It is worth mentioning that all other information sources are fed to the EKF with the exception of the iUSBL observations.

By analyzing the results from Figure 10, it can be seen that innovation peaks are located around zero, resembling a zero-mean Gaussian distribution. This is a positive sign that all observations are uncorrelated, which is a good indication of the performance of the EKF. However, the maximum error $\xi_{max}$ was around 0.4 m in Beacon 1 and Beacon 3, while Beacon 2 showed a smaller error, below 0.3 m. The maximum error occurs during the launching stage, mainly due to incorrect initialization of the speed of sound. Since Beacon 2 stands closer to the MV in the deployment phase, a smaller error is expected.

**Figure 11:**
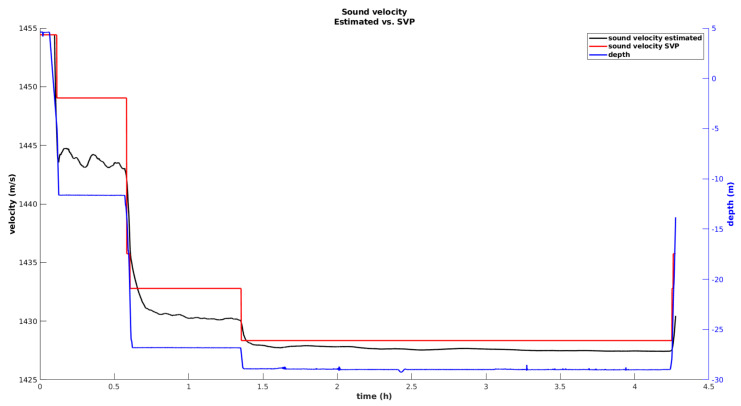
Comparison between the estimated sound velocity estimated and the SVP measurements along the water column.

The evolution of the speed of sound during the dive is represented in Figure 11. A direct comparison with data from an external SVP is established. As the MV goes down through the water column, a variation in the speed of sound is visible, with a maximum variation of approximately 1.8% registered in this trial. At 1 m from the surface, the SVP measures a sound speed of around 1454 m/s, while at 29 m, the sound speed drops to 1428 m/s.

Initially, a substantial error, between the SVP and sound velocity state, estimated by the filter, is observed. This error affects the range observations, originating higher range innovations, as discussed previously. Nevertheless, the filter catches up, and the error decreases. However, a persistent offset, of around 0.07% (1 m/s in 1428 m/s), is visible during the entire cutting phase. Some component of this offset can be associated with the calibration of the SBL array, which is performed by placing a GNSS receiver on top of the pole of each transponder. Despite the high accuracy of this measurement, the pole’s orientation introduces some uncertainty, which is reflected in a deviation between the true beacon position and the one determined through calibration. Since the calibrated position is necessary to calculate the predicted observation (Equation (15)), we suspect that a calibration error component is being compensated by over correcting the speed of sound state. For example, over a range of 50 m, a calibration error of 3.5 centimeters can be interpreted as a variation of 1 m/s in the speed of sound. Nevertheless, it is necessary to consider that the comparison established between the SVP and the estimated speed of sound is not completely fair, as the SVP provides a local measurement, while the state estimate corresponds to the average speed of sound through the water column. However, this comparison is useful as it demonstrates that, in terms of variation with respect to depth, both methods show a similar temporal variation. This is an indication that the speed of sound is being correctly observed by the filter.

**Figure 12:**
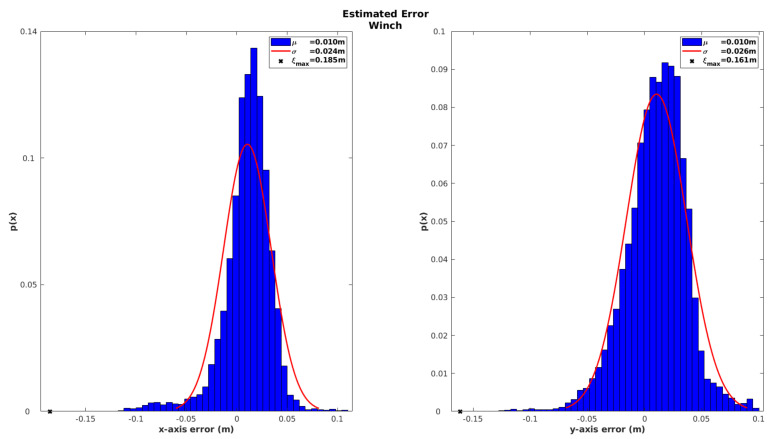
Absolute position error of the SBL range-only model characterized by winch innovations.

Finally, in order to characterize the results of the localization solution based only on SBL ranges, the innovation of the winch observation is analyzed in Figure 12.

As the pressure sensor provides direct depth observation with high accuracy, the results based on winch innovations focus only the XY-axes. Results from Figure 12 show a maximum error in the X-axis of around 0.185 m and 0.161 m for the Y-axis, with a standard deviation below 0.048 m. This result demonstrates the high accuracy of our localization solution even when considering only SBL acoustic measurements. It also demonstrates the capability to perform the recovery maneuver in a very precise way.

### 4.2. USBL Model Only

As previously mentioned, the close proximity between array elements makes USBL devices more prone to multipath reflections and occlusions, when compared with acoustic positioning systems with larger baselines. Therefore, a data association approach was implemented for outlier rejection, ensuring that only coherent measurements are introduced in the data fusion stream. Our method exploits the directional information intrinsic to the iUSBL measurements to correct drift affecting mainly the heading angle.

**Figure 13:**
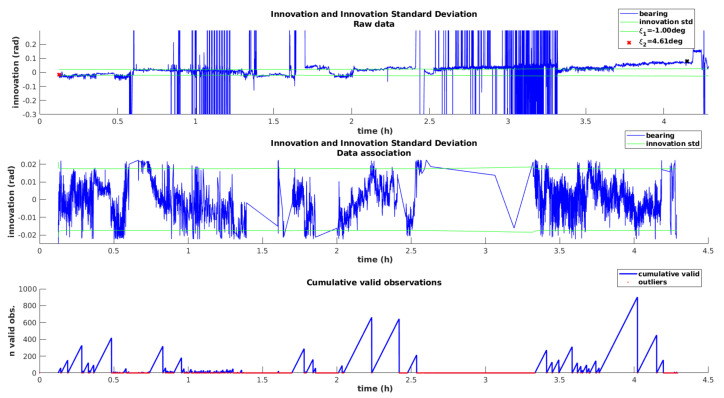
Performance analysis of the iUSBL observation model and data association method. (**Top**) Simulation of the innovation for the bearing observations, considering iUSBL observations with outliers. (**Middle**) Real innovation sequence obtained by introducing bearing observations in the data fusion stream and by performing data association. (**Bottom**) Cumulative representation of the consecutive observations accepted by the outlier rejection method.

Figure 13 demonstrates the performance of this approach. The first plot simulates the innovation sequence for the bearing angle, as well as the innovation standard deviation, while using the raw measurements directly, without performing the outlier rejection method. Please note that these raw measurements are not fed to the filter at this point. The purpose of this representation is to demonstrate the high number of outliers, which are clearly visible by the spikes in the innovation sequence. This plot also illustrates the drift affecting the heading angle, as the innovation sequence deviates from zero with time.

The second plot from Figure 13 represents once again the bearing innovation and corresponding standard deviation, but this time, iUSBL measurements are being supplied to the filter and the data association method is running. A clear improvement is visible, since the innovation fits inside the standard deviation and outlier spikes were completely removed. The zero-mean innovation behavior indicates that the heading drift is being effectively mitigated, through the inclusion of orientation observations from the iUSBL system. As shown by the third plot, which represents the number of consecutive valid measurements, a high percentage of iUSBL observations is rejected by the data association method.

**Figure 14:**
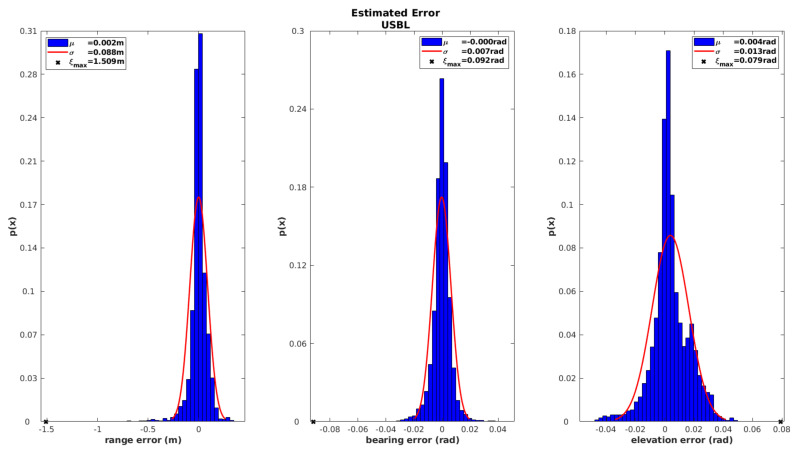
Histograms of the innovation sequence for the iUSBL observation model, defined in terms of range and directional angles.

The analysis of the iUSBL observation model should also be performed in terms of the elevation angle and range. Figure 14 summarizes the behavior of the innovation sequence for the three observed parameters. Results from Figure 14 show a Gaussian shape with a zero mean for all the cases, which indicates a good performance. However, the maximum error $\xi_{max}=1.5$ m obtained for the range observation is much higher than the results obtained for the SBL range-only model, which indicates that the positioning performance of the SBL system is superior to the one achieved by the iUSBL system.

**Figure 15:**
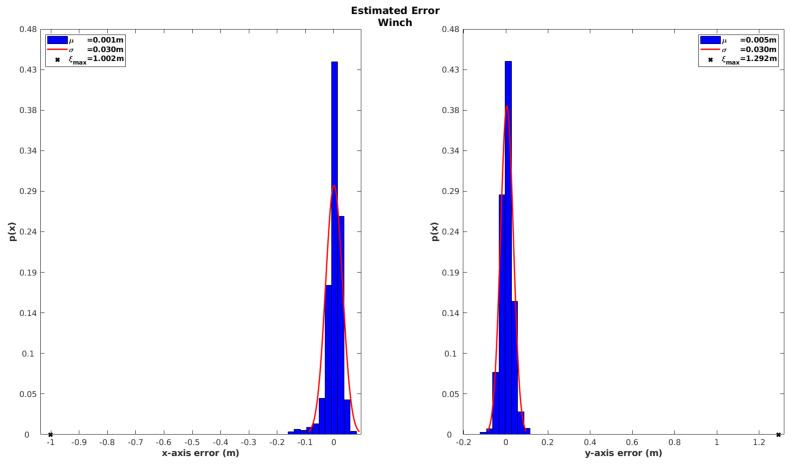
Absolute position error for the USBL model characterized in terms of the innovation of the winch observation.

Once again, in order to assess the global consistency of the localization solution achieved by the iUSBL observation model, the innovation sequence of the winch observation is analyzed during the recovery maneuver in Figure 15. The histograms show a zero-mean Gaussian shape for the winch innovation, indicating a good performance for this observation model. When compared with the results obtained for the SBL range-only observation model (see Figure 12), we can conclude that the iUSBL system performs slightly worse in terms of positioning. Nevertheless, the iUSBL has the great advantage of providing directional information for correcting attitude drift.

### 4.3. SBL and USBL Models

**Figure 16:**
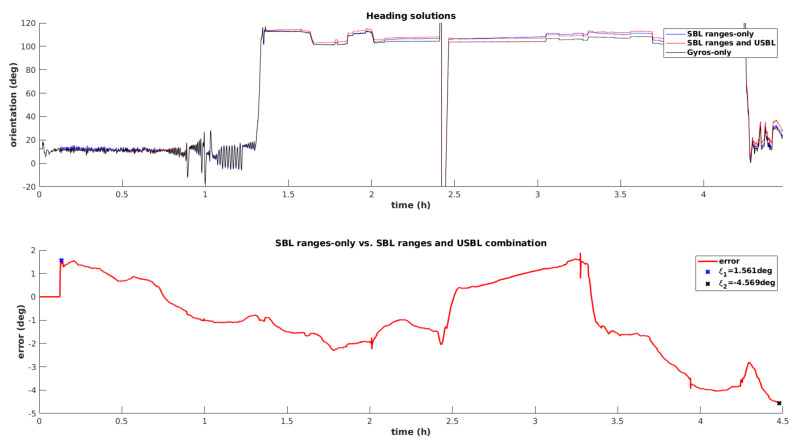
Evaluation of the heading angle estimation using all observation models.

Finally, the data fusion filter is tested using both observation models simultaneously. The first plot from Figure 16 establishes a comparison in terms of the estimated heading angle computed based on three solutions, namely the dead reckoning method, using the gyroscope measurement exclusively, the dead reckoning aided by the SBL range-only model, and dead reckoning plus range-only observations and iUSBL measurements. It can be conclude that combining range-only observations with dead reckoning do not introduce a clear advantage. On the contrary, the inclusion of directional measurements, obtained through the iUSBL observation model, effectively corrects drift affecting the heading angle. The bottom plot from Figure 16 represents the difference between the dead reckoning solution and the one obtained by the integration of the iUSBL measurement model. It can be seen that both solutions differ considerably at the end of the mining mission. The tendency slope, clearly identified in the bottom plot, corresponds to the error drift associated with the dead reckoning solution. The initial error $\xi_{1}$ is explained by a wrong initialization of the heading angle by the north seeking method. However, the purposed solution converges after receiving updates from the iUSBL system. It can be seen that after 4 h and 30 min, the dead reckoning heading solution deviates more than 4.5 degrees.

**Figure 17:**
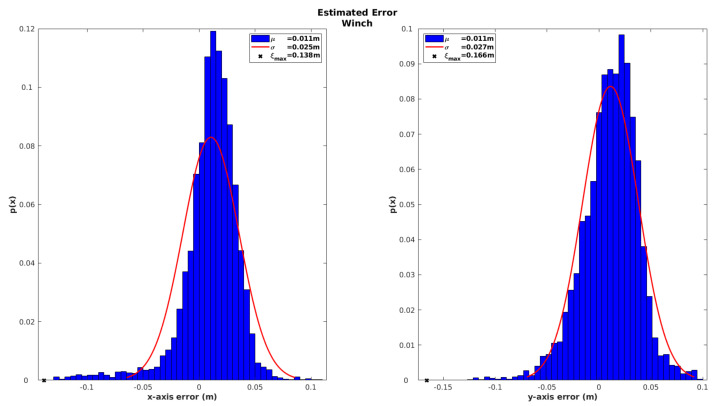
Absolute position error obtained for the two observation models combined, assessed through the innovation sequence of the winch observation.

The advantage of combining both observation methods becomes evident when analyzing the performance in terms of position. In this case, the innovation of the winch observation, depicted in Figure 17, demonstrates a considerable accuracy improvement on the estimated horizontal position, when combining both SBL and iUSBL measurements. By supplying all available measurements to the EKF, a global positioning error with less than 3 cm of standard deviation is achieved.

## 5. Conclusions

Results presented in this paper corroborate the evidence experienced on-site regarding the consistency of the localization solution. In the field, operators were able to remotely control the mining vehicle based exclusively on the virtual reality representation.

Individual results were presented in order to clearly demonstrate the improvements achieved by each of the purposed observation models. Fusing individual range measurements, as opposed to the traditional SBL multilateration technique, provides a clear advantage in terms of robustness and flexibility. This approach enables the integration of range observations even when only a single beacon receives the acoustic reply, while traditional solutions require that the response is received by at least three beacons. Therefore, periods without observations are reduced, resulting in lower drifts produced by the dead reckoning strategy alone. This approach is also more robust since it is possible to apply outlier rejection techniques to individual range measurements. This is beneficial since one observation may be rejected without discarding all other observation computed by the remaining SBL beacons. Data association was demonstrated for the iUSBL case, with noticeable success on rejecting outliers and thus providing consistent directional observations, to accurately compensate the gyroscope bias.

In the future, some improvements will be developed to attain a higher localization accuracy. The extrinsic position of each beacon will be incorporated in the localization state vector, and thus, better results can be achieved.

## Figures and Tables

**Table 1:** Technical specifications associated with the IMU, installed on board the MV, and the acoustic positioning transceivers and the GNSS receivers, installed on the LARV.

KVH 1775 FOG IMU	
**Gyroscopes**	
Input Rate (max)	±490°/s
Bias Instability @ 25°	≤0.1°/h, 1σ (max)
Bias Offset @ 25°	≤0.1°/h, 1σ (max)
Angle Random Walk @ 25°	≤0.012${}^{\circ}/\sqrt{h}$
**Accelerometers**
Input Limit (max)	±10 g
Bias Instability (constant temperature)	≤0.05 mg, 1σ
Bias Offset	0.5 mg
Velocity Random Walk @ 25°	0.12 mg/$\sqrt{\mathrm{Hz}}$
**Magnetometers**
Input Range	±10 Gauss
Bias	<0.2 Gauss
Bias Noise (rms)	<2 mGauss
**Update rate**	≤5000 Hz
**Evologics S2C R 42/65 Transceivers**	
Operating Range	<2000 m
Slant Range Accuracy	0.01 m
Bearing Resolution (USBL only)	0.1 deg
Acoustic Connection	up to 31.2 kbit/s
Update Rate	≤1 Hz
**Unicore UB482 GNSS**	
Constellations	GPS, GLONASS, GALILEO, BDS
Frequencies	L1 L2 B1 B2 E1 E5b
RTK (rms)	horizontal: 1 cm + 1 pm vertical: 1.5 cm + 1 pm
Heading (rms)	0.2 degree/1 m baseline
Update Rate	≤20 Hz

## Abbreviations

The following abbreviations are used in this manuscript:

AUV	Autonomous underwater vehicle
CC	Control cabin
DR	Dead reckoning
DVL	Doppler velocity log
EKF	Extended Kalman filter
FOG	Fiber optic gyroscope
GNSS	Global navigation satellite system
IMU	Inertial measurement unit
iUSBL	Inverted ultrashort baseline
KF	Kalman filter
LARV	Launch and recovery vessel
LBL	Long baseline
MV	Mining vehicle
PF	Particle filter
RTK	Real-time kinematic
SBL	Short baseline
SVP	Sound velocity probe
TOF	Time of flight
UKF	Unscented Kalman filter
USBL	Ultrashort baseline
UUV	Unmanned underwater vehicle
